# Social Exclusion and Green Consumption: A Costly Signaling Approach

**DOI:** 10.3389/fpsyg.2020.535489

**Published:** 2020-11-04

**Authors:** Yulang Guo, Pan Zhang, Junyun Liao, Fang Wu

**Affiliations:** ^1^School of Business Administration, Guangdong University of Finance and Economics, Guangzhou, China; ^2^Business School, Sichuan University, Chengdu, China; ^3^School of Management, Jinan University, Guangzhou, China; ^4^School of Accounting, Jiangxi University of Finance and Economics, Nanchang, China

**Keywords:** green consumption, social exclusion, Costly Signaling Theory, purchasing occasion, exclusion attribution

## Abstract

This article is dedicated to examine the impact of social exclusion (i.e., being rejected, isolated, excluded or ignored by other individuals or groups in society) on consumers’ intention of green consumption. Based on Costly Signaling Theory, three experiments have been conducted to explore one main effect and the corresponding mechanism together with two boundary conditions. Specifically, the first study tests the main effect and internal mechanism by manipulating the state of social exclusion. The results show that social exclusion enhances consumers’ intention to buy green products and consumers’ desire for self-sacrifice mediates that relationship. Study 2 manipulates audience state to examine the first boundary condition. The findings show that the effect of social exclusion on green consumption exists only in public purchasing scenarios. Study 3 tests the second boundary condition by manipulating the stability of exclusion causes. The results indicate that the main effect is significant only when causes of exclusion are not stable. The final part discusses theoretical contributions and practical implications of this study in the field of both social exclusion and green consumption.

## Introduction

Social exclusion is common in modern society. It takes place in situations when one is discriminated at school, treated unfairly in the workplace, ignored by salespersons, rejected when adding friends or initiating chat interactions on the Internet (Mead et al., 2011; Duclos et al., 2013; Pieters, 2013; Ward and Dahl, 2014). Although the impact of social exclusion on consumer behavior has received increasing attention in recent studies (Mead et al., 2011; Duclos et al., 2013; Pieters, 2013; Ward and Dahl, 2014), very little is known about how social exclusion affects consumers’ prosocial behavior like green consumption. As an important form of prosocial behavior, green consumption is an effective approach to mitigate the negative effect of human activities on the natural environment. However, as green goods are usually more expensive and with lower quality than their non-green counterparts (Dietz et al., 2003; Griskevicius et al., 2010), consumers’ passion for green goods is dampened to a certain extent. Moreover, conclusions about the impact of social exclusion on prosocial behavior are not consistent in the existing literature. Some studies find that socially excluded people are willing to conduct prosocial behavior to regain social belonging (Mead et al., 2011; Lee and Shrum, 2012), while other evidence shows that social exclusion can retard prosocial behavior (Twenge et al., 2007). Social exclusion can cause empathy shortage and weaken self-management capability (Twenge et al., 2003, 2007), which may discourage consumers from buying green products. Therefore, the relationship between social exclusion and green consumption is waiting to be clarified.

### Social Exclusion and Its Consequences

In recent years, social exclusion has attracted wide attention from scholars of consumer behavior. It refers to subjective feelings when one is rejected, isolated, excluded or ignored by other individuals or groups in society, in which situation his or her needs of belonging and social interaction cannot be met (Williams, 2007). The need for social relations is one of the basic needs for human being, while social exclusion is a direct threat to it. Many studies show that social exclusion has a significant negative impact on individual behavior. For example, social exclusion can stimulate pain-related brain areas to be more active (Eisenberger et al., 2003), and weaken individuals’ self-management capability (Baumeister et al., 2005). As for consumer behavior, when suffering from social exclusion, consumers will perceive that their needs of belonging are threatened (Mead et al., 2011), and then make more conformity consumption and show stronger preferences for material products. In addition, socially excluded consumers prefer luxury goods, and have a stronger risk-seeking tendency in financial decision-making (Mead et al., 2011; Duclos et al., 2013; Pieters, 2013; Ward and Dahl, 2014).

### Green Consumption and Its Antecedents

Green products refer to products related to environmental conservation, including organic products, energy-saving products, and products free of chemically toxic substances. Accordingly, green consumption refers to the fact that consumers take the corresponding impact on the environment into consideration when they buy, use or dispose goods, in order to reduce potential pollution and maximize long-term benefits (Carlson et al., 1993), like buying small and energy-efficient cars instead of high-energy-consumption ones, recycling and reusing waste goods rather than throwing them away. Although consumption of green products is beneficial to the environment, it costs more to pay higher prices for low-performance products and change the original mode of consumption. In this way, green consumption is a manifestation of altruistic or prosocial behavior (Lee and Holden, 1999). However, a gap exists between attitude and behavior in green consumption. Existing studies explored influencing factors and intervention strategies of green consumption from three perspectives. First, from the perspective of environmental conservation, the main reason for people to purchase green products is to protect the earth and its habitats. An effective strategy is to arouse people’s awareness of environmental protection by presenting facts of environmental deterioration, so as to trigger consumers’ green consumption behavior (Owens, 2000). This group of research focuses on the positive role of psychological factors, including consumer motivation, values, environmental concern, subjective norms, and innovation (Follows and Jobber, 2000; Griskevicius et al., 2010; Lin and Chang, 2012) and external intervention strategies like firm publicity information, economic incentives and regulations of governments and enterprises (Nyborg et al., 2006; Goldstein et al., 2008). Second, from the perspective of economic rationality, the cost added is stressed. Emphasizing higher cost-effectiveness of green products in advertisements and information beneficial to consumers, such as tax deductions and being good for health, can help enhance their willingness to buy green products (Van Vugt et al., 1995; Matsukawa et al., 2000). Third, others’ prosocial motivation plays an important role in promoting green consumption intention. Specifically, among the measures adopted to motivate hotel guests to reuse towels, conveying information about other guests’ environmental conservation is more effective than caring for the environment and economic needs. Hence, social orientation factors like reputation are also driving forces of people’s participation in environmental conservation (Van Vugt, 2009).

### Social Exclusion and Green Consumption

In this study, Costly Signaling Theory is applied to examine the effect of social exclusion on green consumption.

#### Costly Signaling Theory

Costly Signaling Theory originates from behavioral ecology. Early studies focused on animal signaling and anthropology with much empirical support (Gurven et al., 2000; Loyau et al., 2005). For instance, peacocks’ beautiful tail signals good genes. To have a beautiful tail, peacocks must consume a lot of food and energy to ensure adequate nutrition on the premise of health. Although it seems wasteful, it conveys a signal that these peacocks have good genes. This theory has been recently applied to the field of psychology (Miller, 2000; Griskevicius et al., 2007). Prior studies show that in public charity, sacrificing time and money can help convey signals about one’s reputation and status. Public display of luxuries and magnanimity is viewed as a form of social competition, in which the most generous, self-sacrificial, or wasteful people win the highest prestige (Bird and Smith, 2005). This theory has also been applied to the context of green products. With conspicuous consumption, consumers can convey signals of social status by purchasing expensive green products (Griskevicius et al., 2010). Prosocial behavior is helpful in social status competition, known as competitive altruism (Hawkes, 1993; Roberts, 1998; Barclay and Willer, 2007; Van Vugt et al., 2007). Purchasing green products is a common altruistic behavior, which takes time, money or other valuable resources for an individual to engage in altruistic activities. Thus, engagement in altruistic behavior indicates adequacy of resources and ability of cost bearing. Meanwhile, it sacrifices personal interests for public welfare. Considering that green products cost more than non-green ones and that socially excluded consumers pay more attention to the signal transmission among social relationships, this study takes Costly Signaling Theory as its theoretical basis.

Specifically, we will compare socially excluded consumers with socially included consumers. Social inclusion refers to subjective feeling when one is accepted, included or valued by other individuals or groups in society. When consumers are excluded from the society, their needs of belonging are threatened and their desire for social connection increases significantly (Maner et al., 2007; Lakin et al., 2008). To regain social connection, consumers are interested in establishing relationships with the surrounding environment (Maner et al., 2007), and then strategically take actions to gain the sense of belonging (Mead et al., 2011). Green consumption is exactly one of these actions. The price of green goods is higher than that of non-green ones. According to Costly Signaling Theory, consumers can send out signals that they have sacrificed enough resources to buy green products (Bird and Smith, 2005). Since green consumption is in line with the long-term benefits of the society, it is easier for consumers to be accepted by other people if they take this action (Griskevicius et al., 2010). Moreover, these signals can also increase the likelihood of their being accepted by other groups. Therefore, socially excluded individuals are more likely to signal their prosociality and wealth by purchasing green products so as to regain the sense of belonging and social connections. Accordingly, the first hypothesis is proposed as follows.

H1: Compared with socially included consumers, socially excluded consumers are more likely to choose green products.

Socially excluded consumers need to seek satisfaction from social connections (Maner et al., 2007; Lakin et al., 2008). Based on Costly Signaling Theory, consumers’ purchasing of green products can send out signals of self-sacrifice for public interests (Bird and Smith, 2005). In doing so, this signal can enable socially excluded consumers to be re-accepted by others. Hence, hypothesis 2 is proposed as follows.

H2: The desire for self-sacrifice mediates the relationship between social exclusion and green consumption.

#### Boundary Condition 1: Audience

According to Costly Signaling Theory, consumers can signal their willingness and ability to be prosocial to the public through green consumption. However, if the signal transmission channel were cut off, the influence of social exclusion would disappear. Many studies show that in public and private purchasing situations, the signaling of resource may have different effects (Griskevicius et al., 2007). Thus, we propose that audience may moderate the impact of social exclusion on green consumption. This variable has two dimensions, public purchase and private purchase. Public purchase means that, in the purchasing process, consumers’ behavior can be seen by or interacted with audiences, like communicating with sales staff and peers. Private purchase refers to personal purchasing behavior with no attention from other people in the process. In public situations, consumers can easily transmit signals to others, including salespersons and their accompanying friends. Therefore, in public purchasing situation, consumers are more sensitive to whether their behavior can be seen and what kind of signals can be transmitted. For socially excluded consumers, they are more likely to buy green products in public to convey the signal of their resource sacrifice for altruistic behavior (Goldberg, 1995; Harbaugh, 1998; Kurzban et al., 2007). On the contrary, in private purchasing situation like sitting alone in front of a computer at home, the signal transmission of green consumption disappears as they cannot regain the sense of belonging and social connections. In this case, altruistic behavior at the expense of their resources becomes not necessary. Consequently, socially excluded consumers are not willing to buy green products. Accordingly, hypothesis 3 is proposed.

H3: In public purchasing situations, socially excluded consumers are more likely to choose green products, while in private purchasing situations, the effect of social exclusion on green consumption will disappear.

#### Boundary Condition 2: Cause of Social Exclusion

The attributions of social exclusion are different, which may affect the relationship between social exclusion and green consumption. Specifically, socially excluded individuals can perceive the stability of reasons for their exclusion. Some causes are stable, while others are unstable (Weiner, 1985). If the cause for exclusion is stable, the individual will deviate from the previous goal of seeking social connections because the status cannot be changed by his or her own efforts (Wan et al., 2014). In this way, there is no need for him or her to transmit the signal through purchasing green products. When the cause is unstable, they can find ways to regain social connections (Wan et al., 2014). At this time, they are willing to send out signals through green consumption, which can help achieve their goals of establishing social connections. Therefore, hypothesis 4 is proposed as follows.

H4: Compared with socially included consumers, socially excluded consumers are more likely to choose green products when the cause of exclusion is unstable. But when the cause of exclusion is stable, the effect of social exclusion on green consumption will disappear.

### The Current Research

In summary, this paper aims to analyze the relationship between social exclusion and green consumption from the perspective of Costly Signaling Theory. Since purchasing green products can signal one’s prosocial nature and ability to afford it, socially excluded people are more likely to be accepted by others through buying these products. This relationship can be mediated by consumers’ desire for self-sacrifice which can help them win appreciation from others so as to mitigate social exclusion. In this case, the effect of social exclusion on green consumption may be stronger when purchasing in public and disappear in private. Moreover, compared with situations when exclusion is attributed to stable causes (e.g., gender), social exclusion can promote more green consumption through consumers’ desire for self-sacrifice when exclusion is attributed to unstable causes (e.g., personal work performance).

This research makes two contributions. Firstly, it identifies social exclusion as an important determinant of green consumption, extending literature on antecedents of green consumption. Secondly, it enriches research on social exclusion. Based on Costly Signaling Theory, this study proposes that social exclusion may induce consumers’ desire for self-sacrifice, and green consumption is an important way to convey the signal of one’s desire for self-sacrifice.

**FIGURE 1:**
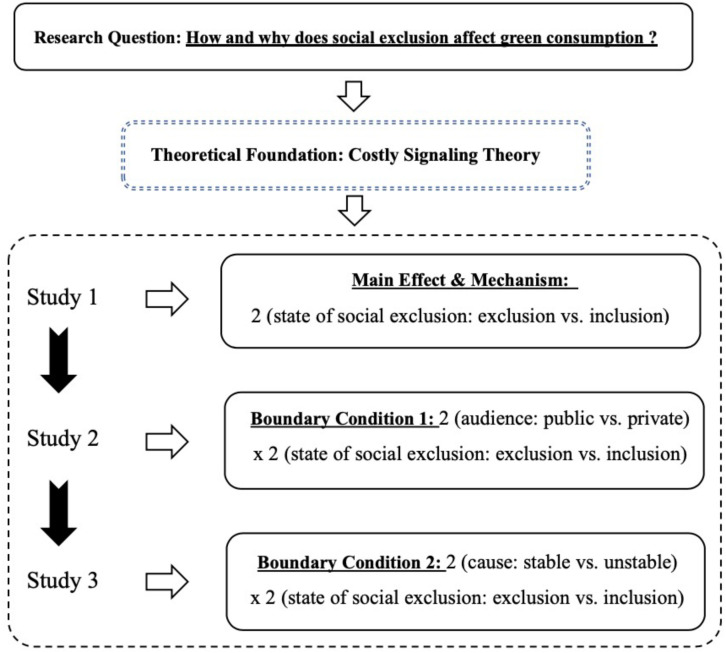
Structure and flow chart of the 3 studies. Figure depicts the structure of this paper. At the first step, the research questions are raised. Then, the theoretical foundation applied to answer research question is proposed. The third part introduces the three empirical studies. Specifically, the first study tests the main effect and mechanism by manipulating the state of social exclusion. The second study examines boundary condition 1 with a between-subjects design. The third study tests boundary condition 2 by manipulating stability of exclusion causes and social exclusion. The mediating variable of desire for self-sacrifice is measured throughout the three studies.

To test the above four research hypotheses, three studies were conducted among which, Study 1 was aimed to test the first two hypotheses, while Study 2 and Study 3 were performed to test the third and fourth hypothesis, respectively. As for the first study, the basic procedure consists of manipulating participants’ state of social exclusion and having them indicate their willingness to buy green product. The following two studies focus on the boundary of Costly Signaling Theory. The key element of Costly Signaling Theory is signal transmission. Study 2 focuses on whether the signal can be successfully transmitted. Consumers cannot send out social signals in private purchasing situations. Study 3 focuses on the necessity of signal transmission. When the cause for exclusion is stable, consumers need not to signal themselves. Specifically, in Study 2, we manipulated audience (whether green goods purchasing can be seen by others), focusing on whether the effect of social exclusion disappears when the signal is not necessary. In Study 3, we manipulated the perceived stability of the cause for exclusion, focusing on whether the effect of social exclusion on green consumption disappears when the signal is not effective. Figure 1 illustrates the structure and flow of the three studies in this research.

## Methods and Results

### Study 1

#### Materials and Methods

The main purpose of Study 1 is to analyze the relationship between social exclusion and consumers’ willingness to buy green products as well as the mediating role of desire for self-sacrifice.

##### Participants

65 marketing major undergraduates (47.7% male, averagely aged 19.46, *SD* = 0.99, 50.8% sophomores and 49.2% juniors) from a university in Western China participated in this experiment in return for CNY 10^1^.

##### Procedures

Participants were randomly assigned to the conditions of a 2 (state of social exclusion: exclusion vs. inclusion) between-subjects design. Social inclusion refers to subjective feeling when one is accepted, included or valued by other individuals or groups in society. It corresponds to the definition of social exclusion. At the beginning of the experiment, the participants were told that they would complete two unrelated tasks.

###### First task: manipulation of social exclusion

The first task was to read a story and imagine them in the context of the story. The story goes that the subject finds three attractive persons on the social network, imagines making a self-introduction, and then sends it to the three persons and asks to make friends with them. Following Rucker et al. (2011), participants in the group of social exclusion were told that their request of making friends was rejected by all of the three persons, while participants in the group of social inclusion were told that their request was approved by all of the three persons.

###### Second task: green consumption and mechanism measure

The second task was to test the participants’ willingness to buy green products. Following Griskevicius et al. (2010), both groups could see two desk lamps on the table with the same price and produced by the same company (see Appendix A). They are labeled as non-green product A and green product B, respectively. Lamp A looks more luxurious, with silk lampshade filtering the best light, and the brightness can be adjusted automatically by induction for 150 Watts. The material of lamp B is more environment-friendly. The lampshade is made with recycled organic fiber cotton cloth, and its energy consumption is only 15% of non-green lamps. The wattage is low but enough for normal use. After reading the materials, participants were asked to fill in the survey of their preference. The endpoint of 1 is the option for non-green product, and 7 for the green product. The larger the number is, the more consumers prefer the green products (*M* = 4.37, *SD* = 0.93). Then, participants were asked to measure their desire for self-sacrifice. The four items come from Kim (2009)’s study, including “I feel good to be able to contribute a little to the public, even if no one pays me for it,” “It is more important to make society better than personal achievement,” “I can make appropriate sacrifices for society better,” and “I hold the belief that fulfilling obligations takes precedence over self-interest,” using Seven Point Likert Scale (*M* = 3.90, *SD* = 0.98, Cronbach’s α = 0.875). Participants were then asked to complete the manipulation check. The item of social exclusion was based on Williams et al. (2000), “In the story experience just now, they felt neglected (or excluded),” with 1 for strongly disagreement, and 7 for strongly agreement. After that, participants were asked to report their emotions with 3 items, including “feeling unpleasant,” “feeling unhappy,” and “feeling bored” (1 = strongly disagreement; 7 = strongly agreement, *M* = 5.38, *SD* = 0.90, Cronbach’s α = 0.847) and complete the basic information (descriptive statistics of variables in three studies see Appendix B). Finally, participants were debriefed, paid and thanked.

#### Results

##### Manipulation check

The results show that participants in the social exclusion group felt more excluded than the social inclusion group [*M*_*social exclusion*_ = 6.50, *SD*_*social exclusion*_ = 1.04 vs. *M*_*social inclusion*_ = 1.68, *SD*_*social inclusion*_ = 0.91, *F*(1,63) = 395.38, *p* < 0.001, $\eta_{\text{p}}^{2}$ = 0.863]. This result suggests that the social exclusion group generally felt more excluded and the manipulation of social exclusion was successful.

##### Willingness to buy green products

**FIGURE 2:**
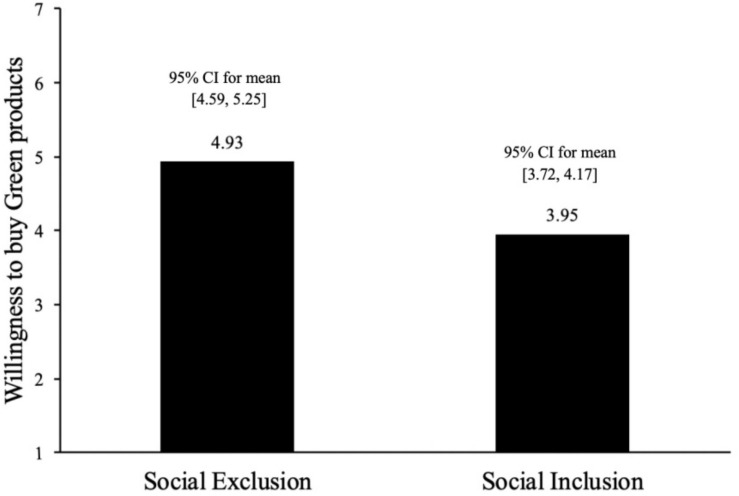
Willingness to buy green desk lamp (Study 1).

The results of ANOVA analysis present the main effect of social exclusion [*F*(1,63) = 24.39, *p* < 0.001, $\eta_{\text{p}}^{2}$ = 0.279] in Figure 2. Compared with the social inclusion group, participants in the social exclusion group showed stronger willingness to buy green products (*M*_social exclusion_ = 4.93, *SD*_social exclusion_ = 0.90, 95% CI for mean [4.59, 5.25] vs. *M*_social inclusion_ = 3.95, *SD*_social inclusion_ = 0.70, 95% CI for mean [3.72, 4.17]; *F*(1,63) = 24.39, *p* < 0.001, $\eta_{\text{p}}^{2}$ = 0.279). Therefore, H1 has been supported. In addition, the results of regression analysis indicated that emotions didn’t have significant effect on the purchase intention of green products (β = 0.029, *t* = 0.254, *p* = 0.800, *r*^2^ = 0.280), which helps rule out the alternative explanation of emotions.

##### Mediating effect test

**FIGURE 3:**
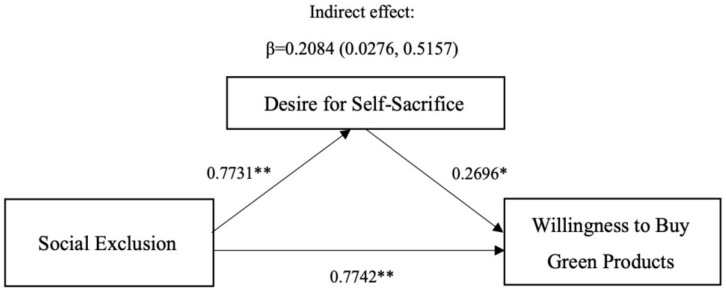
Mediation analysis: desire for self-sacrifice as a mediator (Study 1). ^∗^Significant at the 0.05 level; ^∗∗^significant at the 0.01 level; n.s., not significant at the 0.05 level.

The results showed that participants in the social exclusion group felt stronger desire for self-sacrifice than the social inclusion group [*M*_social exclusion_ = 4.34, *SD*_social exclusion_ = 0.97 vs. *M*_social inclusion_ = 3.57, *SD*_social inclusion_ = 0.86, *F*(1,63) = 11.50, *p* < 0.01, $\eta_{\text{p}}^{2}$ = 0.154]. In order to test the mediating effect, we followed the procedure proposed by Zhao et al. (2010) and the model by Preacher et al. (2007) and Hayes (2013) to conduct the mediating analysis by bootstrapping using 5,000 samples (PROCESS model 4; Hayes, 2013). First, we regressed desire for self-sacrifice on social exclusion. The results testified a significant main effect (β = 0.773, *t* = 3.392, *p* = 0.001, *r*^2^ = 0.392). Second, we regressed willingness to buy green products on social exclusion and desire for self-sacrifice. The results confirmed a significant main effect of desire for self-sacrifice (β = 0.260, *t* = 2.557, *p* = 0.013, *r*^2^ = 0.5898). In sum, the above results support the proposed mediating effect. The indirect effect of the overall sequential mediation model was significant (indirect effect = 0.2084, 95% bias-corrected *CI* ranged from 0.0276 to 0.5157), see Figure 3. Consequently, H2 has been supported.

#### Discussion

Study 1 provided initial evidence for the positive relationship between social exclusion and consumers’ willingness to buy green products, and the mediating effect of desire for self-sacrifice. Based on the Costly Signaling Theory, social exclusion can promote individuals to consume more resources and undertake greater self-sacrifice, thus send out signals to reverse the unfavorable image in the eyes of others. Green consumption is a good way to transmit signals. Study 1 provided evidence for this conjecture and ruled out the explanation of emotions. Social exclusion often leads to negative emotions, and emotions may affect the purchase intention of green products. Although the first study has ruled out this explanation, it did not explore the boundaries of the main effect. According to the Costly Signaling Theory, the premise of this effect is that signals can be transmitted successfully. The following studies will be conducted to test this premise.

### Study 2

#### Materials and Methods

In Study 1, we didn’t discuss the boundary conditions of the main effect. Based on this limitation, the moderating role of audience will be examined in Study 2 to clarify the boundary of the effect of social exclusion on green consumption.

##### Participants

135 economics major undergraduates (46.7% male, averagely aged 20.17, *SD* = 1.09, 54.7% sophomores and 45.3% juniors) from one university in Southern China participated in the experiment in return for CNY 10.

##### Procedures

Participants were randomly assigned to the conditions of a 2 (state of social exclusion: exclusion vs. inclusion) × 2 (audience: public vs. private) between-subjects design. Before the experiment, the participants were told that they would complete two unrelated tasks.

###### First task: manipulation of social exclusion and audience

The first task was to read a story and imagine them in the context of the story. Following Wan et al. (2014), the story describes the basic information of IWE CLUB, a community of foreign high-end-brand game players where members can enjoy value-added services (see Appendix C). Then, participants were asked to imagine submitting a membership application to that community. Those in the social exclusion group were told that the community had rejected their application, while those in the social inclusion group were told that the community had approved their application. The second task was to test the participants’ willingness to buy green products. Firstly, the audience was manipulated. Following Griskevicius et al. (2010), participants in the public group were guided to imagine shopping in a mall, while those in the private group buying online at home alone.

###### Second task: green consumption and mechanism measure

Secondly, participants were asked to read experimental materials of two kinds of tissues (see Appendix D). One group read material of the tissue from company A, with the product doubly flexible, skin friendly, 200 extractions per bag, and not easy to break when being wet. The price is RMB 4.9 per bag. The other group read that of the tissue from company B, made of straw pulp and with no chemical addition, greener, healthier, non-bleaching, more environmentally friendly, 200 extractions per bag. The price is RMB 6.9 per bag. After reading the product information, the subjects were asked to fill in their preference. The endpoint of 1 is option for product A, and 7 for product B. The larger the number is, the more consumers prefer the green product (*M* = 4.56, *SD* = 0.86). Participants were then asked to measure their willingness to sacrifice. Similar to Study 1, items of self-sacrifice willingness come from Kim (2009) (*M* = 3.90, *SD* = 1.26, Cronbach’s α = 0.899). After that, participants were asked to fill in the manipulation check of social exclusion, “In the just story experience, they felt neglected (or excluded).” 1 indicates strong disagreement, and 7 strong agreement. Then, participants were asked to report their emotions (as in Study 1, 1 = strong disagreement; 7 = strong agreement, *M* = 5.64, *SD* = 0.98, Cronbach’s α = 0.883) and complete their basic information. Finally, participants were debriefed, paid and thanked.

#### Results

##### Manipulation check

The results showed that participants in the social exclusion group felt more excluded than the social inclusion group [*M*_social exclusion_ = 6.47, *SD*_social exclusion_ = 1.10 vs. *M*_social inclusion_ = 1.49, *SD*_social inclusion_ = 0.90, *F*(1,133) = 819.02, *p* < 0.001, $\eta_{\text{p}}^{2}$ = 0.860]. That is to say, the social exclusion group generally felt more excluded and the manipulation of social exclusion was successful.

##### Willingness to buy green products

**FIGURE 4:**
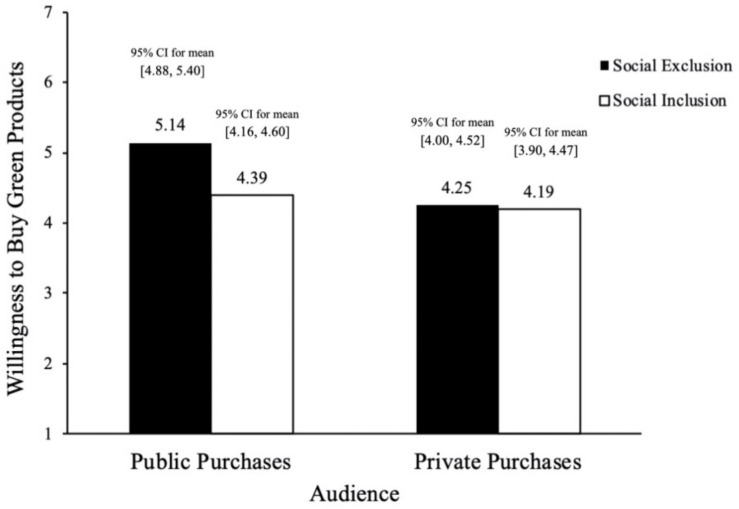
Willingness to buy green tissue (Study 2).

In a 2 × 2 ANOVA, we found that both the main effect of social exclusion [*F*(1,131) = 8.60, *p* < 0.001, $\eta_{\text{p}}^{2}$ = 0.062] and the interaction effect of social exclusion and audience [*F*(1,131) = 6.28, *p* < 0.01, $\eta_{\text{p}}^{2}$ = 0.046] were significant, see Figure 4. We also carried out a simple effect analysis. In public purchasing situations, compared with the social inclusion group, participants in the social exclusion group showed stronger willingness to buy green products (*M*_social exclusion_ = 5.14, *SD*_social exclusion_ = 0.87, 95% *CI* for mean [4.88,5.40] vs. *M*_social inclusion_ = 4.39, *SD*_social inclusion_ = 0.75, 95% CI for mean [4.16, 4.60]; *F*(1,131) = 6.28, *p* < 0.001, $\eta_{\text{p}}^{2}$ = 0.136). In private purchasing situations, participants didn’t differ in their willingness to buy green products (*M*_social exclusion_ = 4.25, *SD*_social exclusion_ = 0.70, 95% *CI* for mean [4.00, 4.52] vs. *M*_social inclusion_ = 4.19, *SD*_social inclusion_ = 0.68, 95% CI for mean [3.90, 4.47]; *F*(1,131) = 0.07, *p* = 0.79, $\eta_{\text{p}}^{2}$ = 0.001). Therefore, H3 has been supported. In addition, the results of regression analysis showed that emotions didn’t have significant effect on the purchase intention of green products (β = 0.116, *t* = 1.675, *p* = 0.096, *r*^2^ = 0.193), which helps rule out the alternative explanation of emotions.

##### Moderated mediation

**FIGURE 5:**
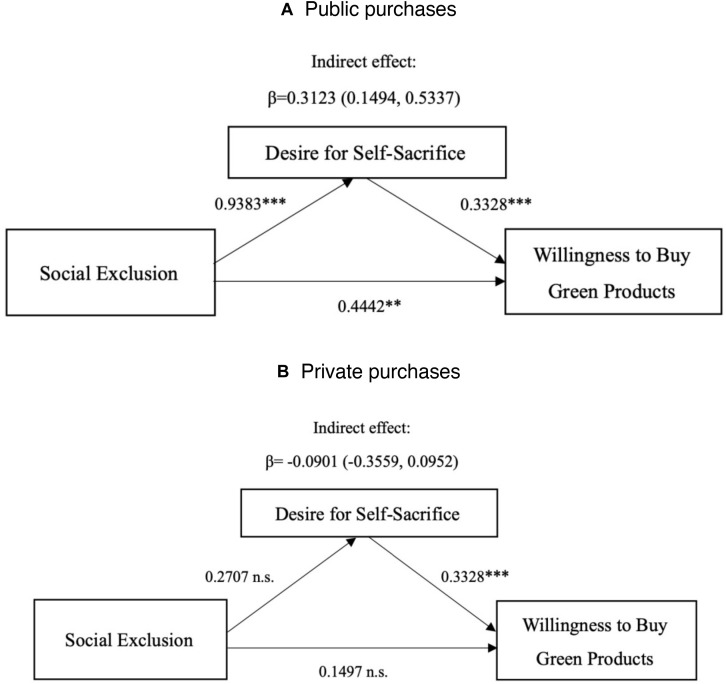
Mediation analysis: desire for self-sacrifice as a mediator (Study 2). ^∗∗^significant at the 0.01 level; ^∗∗∗^significant at the 0.001 level; n.s., not significant at the 0.05 level.

The results showed that participants in the social exclusion group felt stronger desire for self-sacrifice than the social inclusion group in public purchase [*M*_social exclusion_ = 4.77, *SD*_social exclusion_ = 1.14 vs. *M*_*social inclusio*__*n*_ = 3.83, *SD*_social inclusion_ = 1.08, *F*(1,131) = 15.82, *p* < 0.01, $\eta_{\text{p}}^{2}$ = 0.108]; participants didn’t differ in desire for self-sacrifice in private purchase [*M*_social exclusion_ = 3.10, *SD*_social exclusion_ = 1.04 vs. *M*_*social inclusio*__*n*_ = 3.37, *SD*_social inclusion_ = 1.08, *F*(1,131) = 0.74, *p* = 0.39, $\eta_{\text{p}}^{2}$ = 0.006]. In order to test the moderated mediation effect of desire for self-sacrifice, the study followed the analyzing procedure proposed by Zhao et al. (2010), and the model by Preacher et al. (2007) and Hayes (2013), and conducted the mediating analysis by bootstrapping using 5,000 samples (PROCESS model 8; Hayes, 2013). First, we regressed desire for self-sacrifice on social exclusion, audience and their interaction. The results confirmed a significant interaction effect (β = −1.209, *t* = −3.068, *p* = 0.003, *r*^2^ = 0.262). Second, we regressed willingness to buy green products on social exclusion, audience, desire for self-sacrifice and social exclusion × audience. The results identified a significant main effect of desire for self-sacrifice (β = 0.333, *t* = 6.096, *p* < 0.001, *r*^2^ = 0.388). The moderated mediation effect of the overall model was significant (moderated mediation effect = −0.4024, 95% bias-corrected *CI* ranged from −0.7883 to −0.1438), supporting moderated mediation effect. Specifically, in public purchase, the indirect effect was significant (indirect effect = 0.3123, 95% bias-corrected *CI* ranged from 0.1494 to 0.5337). In private purchase, the indirect effect was not significant (indirect effect = −0.0901, 95% bias-corrected *CI* ranged from −0.3559 to 0.0952), see Figure 5. H3 was supported.

#### Discussion

Study 2 replicated the findings of Study 1 by manipulating social exclusion in another method. Furthermore, in order to clarify of the boundary of Costly Signaling Theory, we examined the moderating role of audience in Study 2. The data shows that social exclusion can enhance willingness to purchase green products through self-sacrifice desire in public purchase. However, in private purchase, the effect is no longer significant, and the mediating effect of self-sacrifice intention is also not supported. These results further validate the Costly Signaling Theory. In the process of green products purchasing, signals must be sent out to the public successfully. Otherwise, consumers will lose their intention of green consumption. This is similar to the basic logic of impression management. A large number of studies show that individuals can influence the perception of other people about a person, object or event by regulating and controlling information in social interaction (Aronson et al., 2009). This study extends existing literature by exploring the role of impression management in the context of social exclusion. In real life, individuals are socially excluded for various reasons. However, in Study 2, we didn’t examine the effect for different types of social exclusion. The types may vary, and these differences may affect consumers’ subsequent cognitive processing.

### Study 3

#### Materials and Methods

Due to limitations of Study 2 in which we did not take different types of social exclusion into consideration, we will examine the moderating role of perceived stability of the cause of exclusion in Study 3 to clarify the boundary of Costly Signaling Theory.

##### Participants

138 English major undergraduates (48.6% male, averagely aged 20.26, *SD* = 1.15, 44.9% sophomores and 55.1% juniors) from a university in Southern China participated in this experiment in return for CNY 10.

##### Procedures

Participants were randomly assigned to the conditions of a 2 (state of social exclusion: exclusion vs. inclusion) × 2 (cause: stable vs. unstable) between-subjects design. Before the experiment, the participants were told that they would complete two unrelated tasks.

###### First task: manipulation of social exclusion and cause

In the first task, following Study 2, participants were asked to read the basic information of IWE CLUB, and then imagine submitting a membership application to the community. The manipulation of social exclusion also followed that in Study 2. The attribution manipulation was based on Wan et al. (2014). Participants in the stable group were told that whether they could gain the approval for their application mainly depended on whether their residence met the requirements of the community. Participants in the unstable group were told that whether they could gain approval mainly depended on whether their residence met the requirements of the current community. Even if they were rejected currently, they might get approval in the near future, since the required areas were gradually expanding.

###### Second task: green consumption and mechanism measure

The second task was to test participants’ willingness to buy green products. We used the same two kinds of tissues as in Study 2 (*M* = 4.54, *SD* = 0.83). Participants were then asked to measure their willingness to sacrifice. Items were the same as those in Study 2 (*M* = 3.79, *SD* = 1.21, Cronbach’s α = 0.898). The items of self-sacrifice desire came from Kim (2009). Participants were asked to complete the manipulation check of social exclusion, “In the just story experience, they felt neglected (or excluded).” 1 indicates strong disagreement, and 7 strong agreement. As for the manipulation check of social exclusion causes, there are three items in the scale, including “My application results can be changed through my efforts,” “It is not difficult to change the application results,” and “It is not easy to change the application results (reverse coding).” Finally, participants were asked to report their emotions (as in Study 1, 1 = strong disagreement; 7 = strong agreement, *M* = 5.62, *SD* = 0.96, Cronbach’s α = 0.878) and completed their basic information and were thanked for their participation.

#### Results

##### Manipulation check

The results showed that participants in the social exclusion group felt more excluded than the social inclusion group [*M*_*social exclusio*__*n*_ = 6.41, *SD*_social exclusion_ = 1.14 vs. *M*_social inclusion_ = 1.43, *SD*_social inclusion_ = 0.72, *F*(1,136) = 944.08, *p* < 0.001, $\eta_{\text{p}}^{2}$ = 0.874]. That is to say, the social exclusion group generally felt more excluded and the manipulation of social exclusion was successful. The results also showed that participants in the unstable group felt that the degree of change was higher than those in the stable group [*M*_*unstable*_ = 6.12, *SD*_*unstable*_ = 0.80 vs. *M*_*stable*_ = 2.16, *SD*_*stable*_ = 0.65, *F*(1,136) = 1012.47, *p* < 0.001, $\eta_{\text{p}}^{2}$ = 0.882]. In other words, the manipulation of cause stability was successful.

##### Willingness to buy green products

**FIGURE 6:**
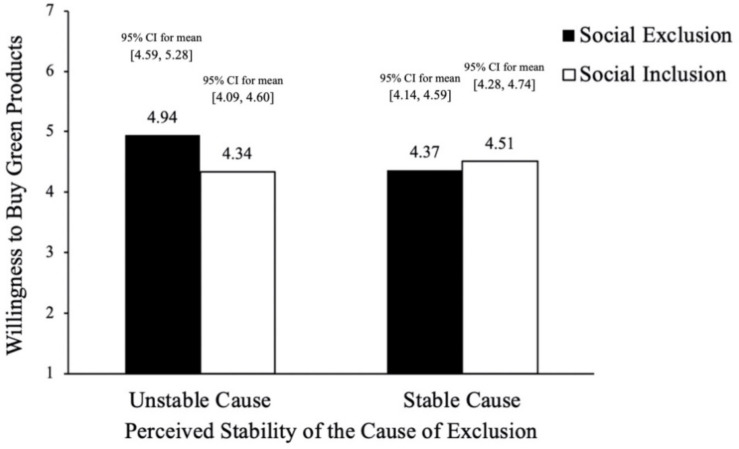
Willingness to buy green tissue (Study 3).

In a 2 × 2 ANOVA analysis, we found significant main effect of social exclusion [*F*(1,134) = 2.79, *P* < 0.001, $\eta_{\text{p}}^{2}$ = 0.020], and interaction effect of social exclusion and stability of cause [*F*(1,134) = 7.41, *p* < 0.01, $\eta_{\text{p}}^{2}$ = 0.052], see Figure 6. We also carried out a simple effect analysis. When the cause of exclusion was unstable, compared with the social inclusion group, participants in the social exclusion group showed stronger willingness to buy green products (*M*_social exclusion_ = 4.94, *SD*_social exclusion_ = 1.03, 95% *CI* for mean [4.59, 5.28] vs. *M*_social inclusion_ = 4.34, *SD*_social inclusion_ = 0.76, 95% *CI* for mean [4.09, 4.60]; *F*(1,134) = 9.80, *p* < 0.01, $\eta_{\text{p}}^{2}$ = 0.068). When the cause of exclusion was stable, participants didn’t differ in willingness to buy green product (*M*_social exclusion_ = 4.37, *SD*_social exclusion_ = 0.69, 95% *CI* for mean [4.14, 4.59] vs. *M*_social inclusion_ = 4.51, *SD*_social inclusion_ = 0.67, 95% *CI* for mean [4.28, 4.74]; *F*(1,134) = 0.55, *p* = 0.46, $\eta_{\text{p}}^{2}$ = 0.004). H4 has been supported. In addition, the results of regression analysis showed that emotions didn’t have significant effect on the purchase intention of green products (β = 0.099, *t* = 1.334, *p* = 0.184, *r*^2^ = 0.048), which helps rule out the alternative explanation of emotions.

##### Moderated mediation

**FIGURE 7:**
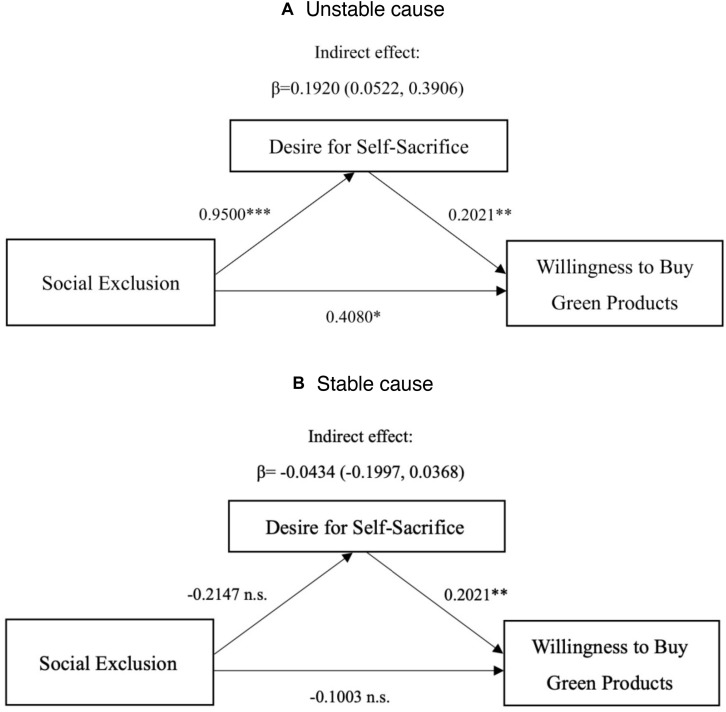
Mediation analysis: desire to self-sacrifice as a mediator (Study 3). *Significant at the 0.05 level; **significant at the 0.01 level; ***significant at the 0.001 level; n.s., not significant at the 0.05 level.

The results showed that participants in the social exclusion group felt stronger desire for self-sacrifice than the social inclusion group, when the cause of exclusion was unstable [*M*_social exclusion_ = 4.76, *SD*_social exclusion_ = 1.13 vs. *M*_*social inclusio*__*n*_ = 3.81, *SD*_social inclusion_ = 1.08, *F*(1,134) = 14.40, *p* < 0.01, $\eta_{\text{p}}^{2}$ = 0.097]; However, participants didn’t differ in desire for self-sacrifice in private purchase, when the cause of exclusion was unstable [*M*_social exclusion_ = 3.17, *SD*_social exclusion_ = 0.97 vs. *M*_*social inclusio*__*n*_ = 3.39, *SD*_social inclusion_ = 1.06, *F*(1,134) = 0.72, *p* = 0.40, $\eta_{\text{p}}^{2}$ = 0.005]. To test the moderating effect of desire for self-sacrifice, this study used the moderated mediation analysis program proposed by Zhao et al. (2010) for reference, and followed the intermediary analysis model (model 8) proposed by Preacher et al. (2007) and Hayes (2013) for bootstrap moderated mediation test. Through bootstrapping 5000, the results showed that desire for self-sacrifice mediates the relationship between social exclusion and the interaction of purchasing occasions and willingness to buy green products. First, we regressed desire for self-sacrifice on social exclusion, cause of exclusion and their interaction. The results confirmed a significant interaction effect (β = −1.165, *t* = −3.265, *p* = 0.001, *r*^2^ = 0.261). Second, we regressed willingness to buy green products on social exclusion, cause of exclusion, desire for self-sacrifice and social exclusion × cause of exclusion. The results identified a significant main effect of desire for self-sacrifice (β = 0.202, *t* = 3.156, *p* = 0.002, *r*^2^ = 0.149). The moderated mediation effect is −0.2354, the confidence interval of Bootstrap test was [−0.5355, −0.0520], and the interval did not contain 0, which indicated that the moderated mediation effect of desire for self-sacrifice was significant. Specifically, in the case of unstable attribution, the indirect effect of self-sacrifice intention was 0.1920, the confidence interval of Bootstrap test was [0.0522, 0.3906], and the interval did not contain 0, see Figure 7. It showed that social exclusion could enhance self-sacrifice intention in the case of unstable attribution, thus affect the purchase intention of green products. Consequently, the direct effect was significant, indicating that desire for self-sacrifice is a partial mediator. Hypothesis 4 has been supported. When the attribution was stable, the indirect effect of self-sacrifice intention was −0.0434, the confidence interval of Bootstrap test was [−0.1997, 0.0368], and the interval contained 0. The data did not support the mediating effect of self-sacrifice intention under attribution stability.

#### Discussion

Individuals are socially excluded for different reasons, so they have different tendencies in their cognitive process, which will affect the boundary effect of this study. In order to further clarify the boundaries of Costly Signaling Theory, we examined the moderating role of attribution types of social exclusion in Study 3. The data shows that social exclusion can enhance purchase intention of green products through desire for self-sacrifice when attribution causes are unstable. However, when attribution causes are stable, the effect is not significant, and the mediating effect of self-sacrifice desire is not supported. These results further validate Costly Signaling Theory. When the causes of social exclusion are stable, even if the signal of self-sacrifice is transmitted through buying green products, it can’t change the state of their social exclusion. This result is consistent with the basic logic of attribution theory. There are differences between external and internal factors in consumer cognitive inference (Anderson, 1983; Weiner, 1985). The stable reasons of social exclusion are equivalent to internal causes, and the unstable reasons equivalent to external causes. When social exclusion is caused by external factors, consumers have a motivation to sacrifice themselves to change others’ views toward them.

## Joint Discussion

This research examines why and how consumers make green purchases in response to social exclusion. From the perspective of Costly Signaling Theory, this research proved that social exclusion has a significant positive impact on consumers’ purchase intention of green products and desire for self-sacrifice plays a mediating role in the relationship (Study 1). Since green products are a good tool for consumers’ signaling of their resource consumption, they are more willing to buy green products after social exclusion. This is consistent with the findings of Griskevicius et al. (2010) that consumers are willing to sacrifice their resources in order to maintain social prestige.

Importantly, two boundary conditions are tested according to Costly Signaling Theory in this paper. The main effect is only significant in public purchase (Study 2). Socially excluded consumers can signal prosociality and resource consumption by purchasing green products so as to meet their needs for social connections. However, the premise of this effect is that the signal can be transmitted successfully. There should be sufficient opportunities to convey information to the audience. In the context of private purchases, social exclusion cannot promote consumers’ willingness to buy green products. In addition, the effect is only significant when the cause of social exclusion is unstable (Study 3). When consumers perceive that the cause for their exclusion is stable, they will give up sending out signals by consuming resources, and they are not willing to buy green products when they are socially excluded. But when consumers believe that the exclusion cause is unstable, they can make efforts to change the status quo and purchase of green products is a good way of signal transmission. The behavior of purchasing products can convey information about one’s consumption of resources. It is conducive to psychological recovery of excluded consumers.

### Theoretical Implications

This study makes three contributions to the literature. Firstly, from the perspective of social exclusion, we examined the influencing factors of green product purchase intention. Previous studies mainly analyzed the antecedents of green consumption from perspectives of environmental conservation and economic rationality. This study explores the relationship from the perspective of social exclusion, enriches the relevant research, and makes a useful supplement to the existing literature. Secondly, social exclusion is very common in our daily life. Previous studies explored its impact on purchase intention of material products, group ownership products and luxury, but not on that of green product consumption. Because of the social value of green products, the integration of the two has certain theoretical significance and enriches social exclusion research in the field of consumer behavior. Thirdly, in previous studies, the mechanisms of social exclusion are mostly the regain of social connections, the need for belonging and the pursuit of uniqueness. Few of them explained the mechanism of social exclusion from the perspective of self-sacrifice. This study confirmed the mechanism of self-sacrifice willingness in the effect of social exclusion on green product purchase willingness through experiments, and provided new theoretical thinking for the mechanism of social exclusion.

### Practical Implications

For marketers, they can enhance consumers’ willingness to buy green products from the perspective of social exclusion. Promoting consumers’ purchase of green products is crucial to construction of ecological civilization and sustainable development of the whole society. In our daily life and purchase scenarios, consumers are often intentionally or unintentionally rejected or ignored. Marketers can seize these opportunities for consumers to be excluded (such as unfair treatment in the workplace, neglected by waiters in restaurants, and setting membership application thresholds) to enhance their willingness to buy green products. Secondly, we should pay more attention to the effect of purchase occasions and attributions of social exclusion on green products purchasing. The influence of social exclusion on green consumption mainly exists on public purchasing occasions and disappears on private purchasing occasions. Online purchasing belongs to private purchasing occasion, and the promotion of social exclusion on the purchase intention of green products disappears. In contrast, most offline purchase situations belong to public occasions. Marketers can develop social exclusion strategies for offline purchase scenarios to promote green consumption. In addition, in the process of product promotion, importance should be attached to conveying information that can change consumers’ self-state through acquired efforts, so that they will feel that they have enough opportunity and are able to change the status quo after exclusion. Thirdly, we should attach more importance to green products’ information transmission function of social values. Consumers buy green products not only because of their willingness to protect the environment and consideration of economic rationality, but also because they can transmit a signal of social values to the outside world. The conclusion of this study shows that consumers transmit the signal of resource cost of self-sacrifice to the public through green consumption. Therefore, marketers should highlight their social values in the design and publicity of green products, such as using green products is the embodiment of their status.

### Limitations and Further Research

There are some limitations in this study waiting to be explored in the future. Firstly, we only used scenario experiments in this study. Although this method has unique advantages in internal validity, its external validity needs to be enhanced. Therefore, other research methods can be utilized to expand its external validity in the future. For example, based on objective data of neuropsychology, the psychological effects of social exclusion and desire for self-sacrifice can be further validated. Secondly, social exclusion is a complex construct. It may have other factors. To be specific, social exclusion can be divided into rejection and neglect. Different types of social exclusion may have different impact on the purchase intention of green products. Thirdly, other mechanisms may exist in the relationship between social exclusion and green consumption. Based on Costly Signaling Theory, this study proposes that desire for self-sacrifice is the internal mechanism. Although the data support our proposition, it is only a partial mediator. Future research may explore other mechanisms at work.

## Data Availability Statement

The datasets generated for this study are available on request to the corresponding author.

## Ethics Statement

The studies involving human participants were reviewed and approved by the Ethics Committee of School of Business Administration, Guangdong University of Finance and Economics, and Human Research Ethics Committee of Business School, Sichuan University. The patients/participants provided their written informed consent to participate in this study.

## Author Contributions

YG designed the research framework. PZ carried out the survey. JL analyzed the data. FW wrote the manuscript. All authors contributed to the article and approved the submitted version.

## Conflict of Interest

The authors declare that the research was conducted in the absence of any commercial or financial relationships that could be construed as a potential conflict of interest.

## Footnotes

1 CNY: Chinese Yuan. CNY 10 ≈ USD 1.47.

## Appendix

(A) Products and product features in Study 1.

**Table:**

**Dowmo’s new lamp in 2019**		
**Dowmo company is a leading enterprise in German lighting industry, which has won various awards in the industry for many years.**		
	**Lamp A**	**Lamp B**

Introduction	Lamp A is made with silk lampshade filtering the best light, and the brightness can be adjusted automatically by induction for 150 Watts.	Lamp B is made with recycled organic fiber cotton cloth, and its energy consumption is only 15% of non-green lamps. The wattage is low but enough for normal use.
Usage scenarios	Work and study	Work and study
Degree of luxury	⋆⋆⋆	⋆
Degree of environment-friendly	⋆	⋆⋆⋆
Price	325RMB	325RMB

(B) Descriptive statistics of three studies.

**Table:**

**Variable**	**No of items**	**Study 1 (*n* = 65)**			**Study 2 (*n* = 135)**			**Study 3 (*n* = 138)**		
		***M***	***SD***	**Cronbach’s alpha**	***M***	***SD***	**Cronbach’s alpha**	***M***	***SD***	**Cronbach’s alpha**
Desire for self-sacrifice	4	3.901	0.982	0.875	3.898	1.259	0.899	3.790	1.205	0.898
Emotions	3	5.385	0.896	0.847	5.637	0.982	0.883	5.616	0.961	0.878
Willingness to buy green products	1	4.369	0.928	N/A	4.563	0.860	N/A	4.544	0.829	N/A

(C) Materials for social exclusion manipulation in Study 2 and Study 3.

About IWE CLUB:

“IWE CLUB” is a 3D game community created by IWE. IWE is one of the most famous high-end game companies in the United States. The company strives to provide players with the best game experience in the world all year round. IWE CLUB provides a communication platform for game players. From today, we will open membership application to some IWE game players. Club members can set up VIP unions in the community, which can add more customization functions to their accounts. They can also play more roles in the game world to improve the game experience.

(D) Products and product features in Study 2 and Study 3.

**Table:**

**You will be faced with a choice of products from two companies in the supermarket where tissue is sold.**		
	**Company A**	**Company B**
Product description	Doubly flexible, skin friendly, 200 extractions per bag, and not easy to break when being wet.	Straw pulp and with no chemical addition, greener, healthier, non-bleaching, more environmentally friendly, 200 extractions per bag.
Degree of cost performance ratio	⋆⋆⋆	⋆
Degree of environment-friendly	⋆	⋆⋆⋆
Price	RMB 4.9 per bag	RMB 6.9 per bag
